# Recovery of genetically defined murine norovirus in tissue culture by using a fowlpox virus expressing T7 RNA polymerase

**DOI:** 10.1099/vir.0.82940-0

**Published:** 2007-08

**Authors:** Yasmin Chaudhry, Michael A. Skinner, Ian G. Goodfellow

**Affiliations:** 1 Calicivirus Research Group, Department of Virology, Faculty of Medicine, Imperial College London, St Mary's Campus, Norfolk Place, London W2 1PG, UK; 2 Vaccine Vector Group, Department of Virology, Faculty of Medicine, Imperial College London, St Mary's Campus, Norfolk Place, London W2 1PG, UK

## Abstract

Despite the significant disease burden caused by human norovirus infection, an efficient tissue-culture system for these viruses remains elusive. Murine norovirus (MNV) is an ideal surrogate for the study of norovirus biology, as the virus replicates efficiently in tissue culture and a low-cost animal model is readily available. In this report, a reverse-genetics system for MNV is described, using a fowlpox virus (FWPV) recombinant expressing T7 RNA polymerase to recover genetically defined MNV in tissue culture for the first time. These studies demonstrated that approaches that have proved successful for other members of the family *Caliciviridae* failed to lead to recovery of MNV. This was due to our observation that vaccinia virus infection had a negative effect on MNV replication. In contrast, FWPV infection had no deleterious effect and allowed the recovery of infectious MNV from cells previously transfected with MNV cDNA constructs. These studies also indicated that the nature of the 3′-terminal nucleotide is critical for efficient virus recovery and that inclusion of a hepatitis delta virus ribozyme at the 3′ end can increase the efficiency with which virus is recovered. This system now allows the recovery of genetically defined noroviruses and will facilitate the analysis of the effects of genetic variation on norovirus pathogenesis.

## INTRODUCTION

Positive-strand RNA viruses of the family *Caliciviridae* are responsible for many diseases in both man and animals. Those that infect humans, namely members of the genera *Norovirus and Sapovirus*, are a major cause of acute gastroenteritis and are thought to be responsible for >85 % of all non-bacterial gastroenteritis outbreaks in Europe between 1995 and 2000 (Lopman *et al.*, 2003).

**Fig. 1. f1:**
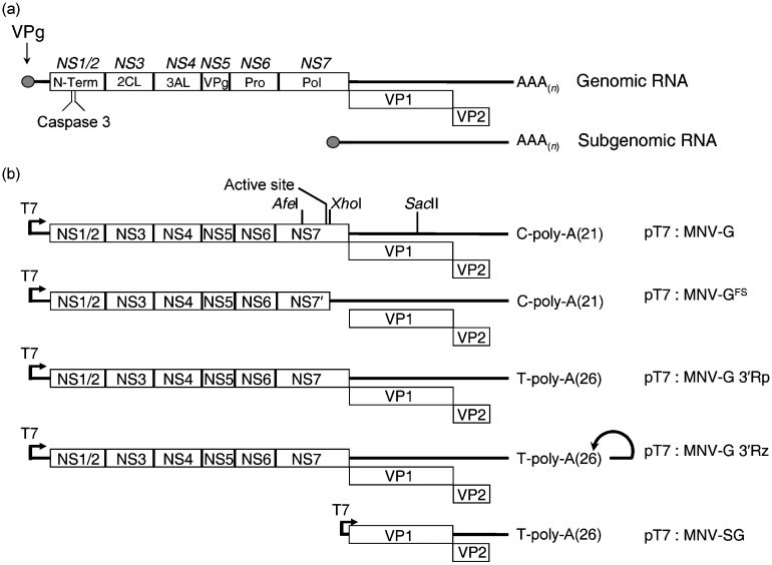
Diagrammatic representation of the MNV genome and the constructs used during this study. (a) Proteolytic-cleavage map of the MNV 1 genome as demonstrated by Sosnovtsev *et al.* (2006). Text in italics highlights the nomenclature as proposed by Sosnovtsev *et al.* (2006). The caspase 3 cleavage sites in NS1/2 and the position of the subgenomic RNA are also indicated. (b) Schematic representation of the constructs used during this study. The positions of the restriction sites used during this study, the active site in the viral RNA-dependent RNA polymerase NS7 and the 3′-terminal nucleotide upstream of the poly-A tail are indicated. See Methods for specific details of each construct.

The calicivirus positive-sense, single-strand RNA genome is approximately 7.5 kb in length, encodes three open reading frames and is linked covalently to the virus-encoded protein VPg (Fig. 1a[Fig f1]). During virus replication, a subgenomic RNA is produced, encoding the major and minor capsid proteins VP1 and VP2, respectively (Fig. 1a[Fig f1]). Despite the major disease burden that caliciviruses cause, we know little with regard to how these viruses replicate and, hence, the identification of potential antivirals and therapeutics has been hindered. This is primarily due to the fact that the human caliciviruses do not replicate efficiently in tissue culture. Although recent reports have demonstrated genome replication and encapsidation by using a vaccinia virus (VACV) expression system (Asanaka *et al.*, 2005; Katayama *et al.*, 2006), the mechanism of synthesis of this RNA and whether it represents authentic VPg-linked viral RNA are still not known. The subgenomic RNA produced from a full-length cDNA clone in one of these systems is not translated, suggesting that it is not VPg-linked (Katayama *et al.*, 2006). A recent report would also suggest that cell lines containing a Norwalk virus replicon can be generated at low frequency (Chang *et al.*, 2006). This lack of an efficient, fully permissive tissue-culture system for human noroviruses has slowed the analysis of the molecular mechanisms used for norovirus genome translation and replication.

To date, functional studies on calicivirus replication have focused on animal caliciviruses, namely feline calicivirus (FCV), porcine enteric calicivirus (PEC; Chang *et al.*, 2002) and the recently identified murine norovirus (MNV) (Karst *et al.*, 2003). The identification of MNV represents a significant advance in the study of norovirus biology, as it replicates efficiently in tissue culture and there is a small-animal model available (Karst *et al.*, 2003; Wobus *et al.*, 2004). The ability to generate genetically defined noroviruses in tissue culture by reverse genetics would provide the first opportunity to understand the relationship between the basic mechanisms of norovirus replication in tissue culture and pathogenesis in a natural host. To date, however, no reverse-genetics system has been reported. Here, we describe a reverse-genetics system that allows the recovery of genetically defined noroviruses in tissue culture. We demonstrate that the traditional VACV-based T7 RNA polymerase delivery system failed to allow the recovery of MNV, due to an inhibitory effect of VACV infection on MNV replication. In contrast, the fowlpox virus (FWPV) T7 RNA polymerase system, which has proved successful for recovery of positive-strand viruses (infectious bronchitis virus; Casais *et al.*, 2001), negative-strand viruses (Newcastle disease virus and rinderpest virus; Peeters *et al.*, 1999; Das *et al.*, 2000) and a bisegmented, double-strand RNA virus (infectious bursal disease virus; Boot *et al.*, 1999), had no deleterious effect on MNV replication and could be used to recover genetically defined MNV in tissue culture.

## METHODS

### Materials.

MNV 1 strain CW1, the murine macrophage line RAW 264.7 and antisera to the MNV VP1 protein were kindly supplied by Herbert Virgin IV (Washington University in Saint Louis, MO, USA). MVA-T7 (Wyatt *et al.*, 1995) was kindly supplied by Bernie Moss (NIH, Bethesda, MA, USA). Rabbit polyclonal antisera to the MNV NS7 RNA-dependent RNA polymerase and VP2 proteins were generated against proteins expressed in and purified from *Escherichia coli* as poly-histidine-tagged proteins (data not shown). Both NS7 and VP2 were purified under denaturing conditions (8 M urea) on nickel agarose and used for immunization of New Zealand White rabbits. All immunizations and test bleeds were carried out by Eurogentec.

All cells were maintained in Dulbecco's modified Eagle's medium (Gibco) containing 10 % fetal calf serum. The Huh 7.5 cell line was kindly provided by Charlie Rice (Rockefeller University, NY, USA). The BSR-T7 cell line was obtained from Karl-Klaus Conzelmann (Ludwig Maximilians University, Munich, Germany).

### Generation of MNV expression constructs.

A full-length cDNA clone of MNV 1 CW1 matching the passage 3 consensus sequence (Wobus *et al.*, 2004), referred to as p20.3 by Sosnovtsev *et al.* (2006), was supplied by Herbert Virgin IV (Washington University in Saint Louis, MO, USA). For clarity, this construct, containing the MNV 1 genome under the control of a truncated T7 RNA polymerase promoter, will be hereafter referred to as pT7 : MNV-G. A derivative of this construct (pT7 : MNV-G^FS^), containing a frame shift in the RNA-dependent RNA polymerase [NS7 in Fig. 1(a)], was generated by linearization with *Xho*I, followed by mung-bean nuclease digestion and religation. A transfer vector containing the *Afe*I–*Sac*II fragment of the MNV-1 genome (pSL301 : MNV *Afe*I–*Sac*II) was generated by cloning the fragment into pSL301 (Invitrogen). To repair the frame-shift mutation in pT7 : MNV-G^FS^, the *Afe*I–*Sac*II fragment from the transfer vector pSL301 : MNV *Afe*I–*Sac*II was inserted into pT7 : MNV-G^FS^ to generate pT7 : MNV-G^FS/R^.

A *Bgl*II restriction site was introduced at position 3959 in the MNV genome by the introduction of a single-nucleotide change (C to A) at position 3959 to generate pT7 : MNV-G/*Bgl*II. The restriction site was introduced by PCR amplification of the region using the primers IGIC44 and 4450R (See Supplementary Table S1, available in JGV Online), digestion with *Afe*I and *Kpn*I and subsequent insertion into the pSL301 : MNV *Afe*I–*Sac*II transfer vector (see above). The mutated fragment was subcloned into pT7 : MNV-G via the *Afe*I and *Sac*II sites. Insertion of the desired mutation, which did not affect the encoded polypeptide sequence, was confirmed by sequencing.

To insert a hepatitis delta virus ribozyme into pT7 : MNV-G at the 3′ end of the genome and to repair the 3′-terminal nucleotide, a derivative of the ribozyme containing a 5′ *Nhe*I site was PCR-amplified from pRZ (Walker *et al.*, 2003) using the primers PUC-F and PUC-R (see Supplementary Table S1, available in JGV Online). The resultant PCR product was digested with *Nhe*I and ligated to the *Nhe*I-digested MNV subgenomic PCR product generated by using primers IGIC21 and IGIC37 (see Supplementary Table S1, available in JGV Online). The ligated product was subsequently digested with *Sac*II and *Eco*RV and inserted into pT7 : MNV-G that had been digested with *Sac*II and *Sna*BI. The resultant plasmid, pT7 : MNV-G 3′Rz, contained the MNV genomic RNA flanked by a truncated T7 RNA polymerase promoter at the 5′ end and a hepatitis delta virus ribozyme at the 3′ end. The RNA produced from this construct contained no additional non-viral nucleotides. A similar construct that lacked the 3′ ribozyme, but which contained a correct 3′-terminal nucleotide, referred to as pT7 : MNV-G 3′Rp, was generated by ligation of the 3′ *Sac*II–*Nhe*I fragment from pT7 : MNV-G 3′Rz with *Sac*II- and *Sna*BI-digested pT7 : MNV. The resulting construct, pT7 : MNV-G 3′Rp, was identical to pT7 : MNV-G 3′Rz, but lacked the 3′ hepatitis delta virus ribozyme.

The MNV 1 subgenomic RNA cDNA expression construct pT7 : MNV-SG was generated by RT-PCR amplification of RNA purified from MNV 1 CW1-infected cells, using primers IGIC21 and IGIC22 (see Supplementary Table S1, available in JGV Online). The amplified product was digested with *Not*I and *Swa*I and inserted into pTriEx1.1 (Novagen) between the *Not*I and *Msc*I sites. The construct was then sequenced fully to confirm that it matched the reported CW1 passage 3 consensus sequence.

### Virus recovery and characterization.

Cells were infected with poxviruses expressing T7 RNA polymerase at an m.o.i. (based on the virus titre in chick embryo fibroblasts) of 0.5–1.0 p.f.u. per cell. Each cDNA expression construct or VPg-linked viral RNA, purified as described previously (Chaudhry *et al.*, 2006), was subsequently transfected (1 μg) by using Lipofectamine 2000 according to the manufacturer's instructions (Invitrogen). To analyse protein expression, cells were harvested 24 h post-transfection for Western blot analysis. To examine the presence of infectious norovirus, cells were harvested 24–72 h post-transfection, virus was released by two freeze–thaw cycles and the titres of virus were determined as TCID_50_ in RAW 264.7 cells, using microscopic visualization for the appearance of cytopathic effect. In some cases, plaque morphology was also examined by plaque assay (Wobus *et al.*, 2004). One-step growth-curve analysis was performed by infecting RAW 264.7 cells at an m.o.i. of 6.0 per cell. Samples were incubated at 37 °C and frozen at −80 °C at various times post-infection. Following two freeze–thaw cycles, the virus titre was determined by TCID_50_ on RAW 264.7 cells.

### Identification of nuclease-resistant, encapsidated MNV RNA.

To remove unencapsidated MNV RNA or excess cDNA, clarified lysates from transfected cells were treated with DNase I (10 units ml^−1^), RNase A (10 units ml^−1^) and RNase T1 (400 units ml^−1^) at 37 °C for 2 h. Encapsidated RNA was partially purified by centrifugation through a 30 % sucrose cushion for 1 h at 55 000 r.p.m. using a Beckman SW55 Ti rotor. The viral pellet was DNase- and RNase-treated prior to extraction of the viral RNA, which used the GenElute system (Sigma). The viral RNA was detected by RT-PCR amplification using the primers 7155F and 7400R (see Supplementary Table S1, available in JGV Online), resulting in the amplification of a 258 bp product.

### Sequence and RT-PCR analyses of recovered viruses.

To determine the sequence of the viruses recovered from cDNA, RAW 264.7 cells were infected with the recovered viruses at an m.o.i. of 3.0 and viral RNA was prepared 24 h post-infection by using the GenElute system (Sigma). Seven overlapping PCR products were amplified, covering the entire genome, and the sequence was determined by using a series of primers (Wobus *et al.*, 2004). To determine the sequence of the 5′ and 3′ ends, 5′ and 3′ rapid amplification of cDNA ends (RACE) was performed by using the Advantage RACE system (Clontech). Sequence analysis and contig generation were performed by using Vector NTI (Invitrogen).

## RESULTS

### VACV infection inhibits MNV replication

**Fig. 2. f2:**
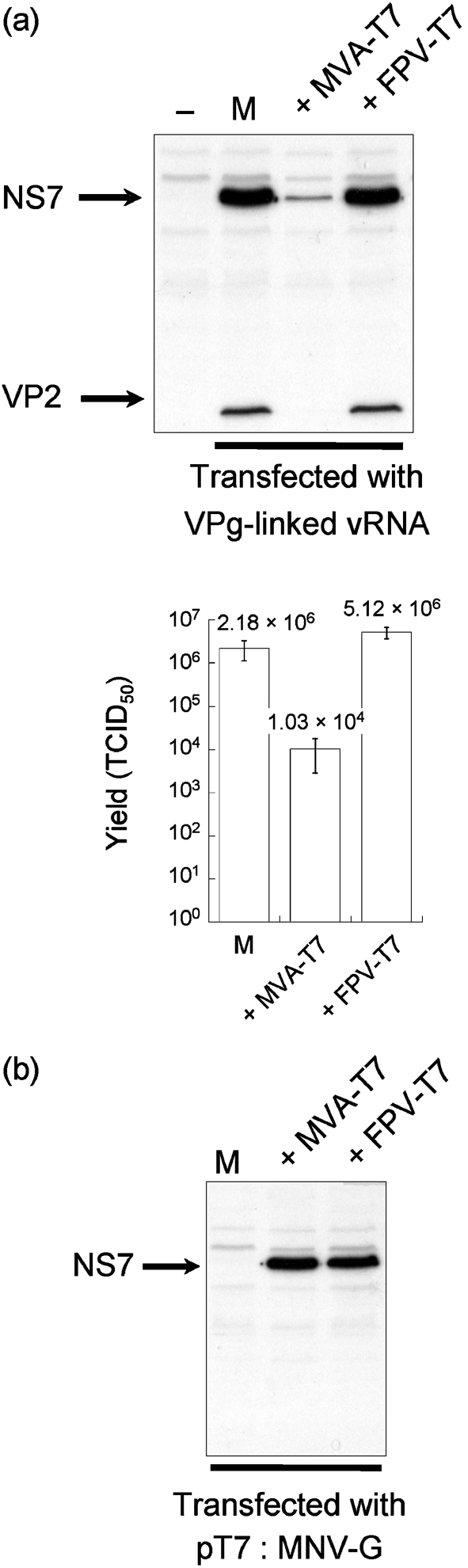
Analysis of the effect of poxvirus infection on MNV replication. (a) BHK cells were mock-infected (M) or infected with either VACV (MVA-T7) or FWPV (FPV-T7) expressing T7 RNA polymerase and subsequently transfected with MNV VPg-linked RNA. Levels of the viral RNA-dependent RNA polymerase (NS7) and minor capsid protein (VP2) were analysed by Western blotting with rabbit polyclonal antisera. In parallel, the virus yield was determined and expressed as TCID_50_ per 35 mm dish. Transfections were carried out in triplicate; error bars represent sd. (b) BHK cells were either mock-infected (M) or infected with MVA-T7 or FPV-T7 and subsequently transfected with a cDNA construct containing the entire MNV genome under the control of a T7 RNA polymerase promoter (pT7 : MNV-G). NS7 expression levels were subsequently analysed by Western blotting.

Previous calicivirus reverse-genetics systems have relied on the transfection of either *in vitro*-transcribed, 5′-capped calicivirus genomic RNA (Chang *et al.*, 2005; Sosnovtsev & Green, 1995) or cDNA constructs, followed by delivery of T7 RNA polymerase using a VACV recombinant (Sosnovtsev *et al.*, 2002; Thumfart & Meyers, 2002). A recent report on rabbit hemorrhagic disease virus (RHDV) has also demonstrated that *in vitro*-transcribed, uncapped RNA is infectious when transfected into cells or delivered directly to the liver *in vivo* (Liu *et al.*, 2006). Attempts to recover MNV by using any of these established approaches failed to produce infectious virus (data not shown). Results indicated that capped, *in vitro*-transcribed MNV RNA is not translated efficiently when transfected into cells (data not shown) and that VACV infection can have a detrimental effect on MNV replication (Fig. 2a[Fig f2]). Levels of the viral RNA-dependent RNA polymerase NS7 and the minor capsid protein VP2 in cells transfected with VPg-linked RNA were reduced significantly when cells had previously been infected with a VACV expressing T7 RNA polymerase (MVA-T7) (Wyatt *et al.*, 1995; Fig. 2a[Fig f2]). This reduction in NS7 and VP2 resulted in a decrease of approximately 200-fold in virus yield (Fig. 2a[Fig f2]). In contrast, prior infection of cells with an FWPV recombinant expressing T7 RNA polymerase (FPV-T7) (Britton *et al.*, 1996) had no effect on NS7, VP2 or virus titre (Fig. 2a[Fig f2]).

A comparative analysis of the ability of T7 polymerase-expressing poxviruses to drive efficient expression of MNV proteins from a cDNA expression construct containing the entire MNV genomic RNA (pT7 : MNV-G; Fig. 1b[Fig f1]) revealed that similar levels of NS7 were synthesized in cells infected with either MVA-T7 or FPV-T7 (Fig. 2b[Fig f2]). The viral capsid proteins VP1 and VP2 were, however, undetectable when cells were transfected with a construct encompassing the MNV genomic RNA (data not shown).

### FWPV expressing T7 RNA polymerase allows recovery of MNV

**Fig. 3. f3:**
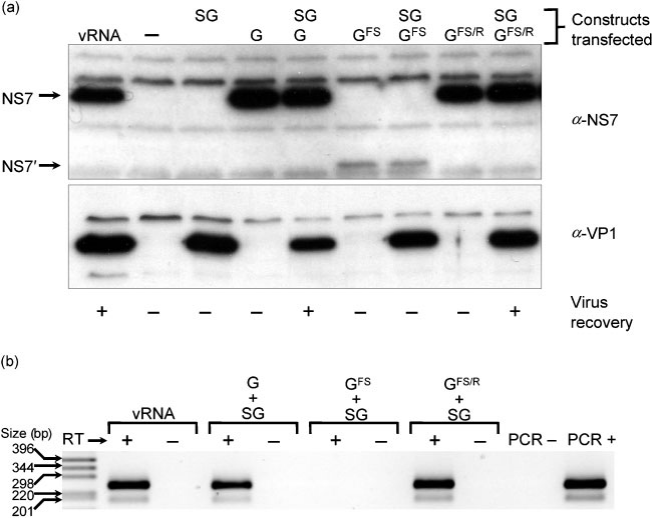
Recovery of genetically defined noroviruses in tissue culture. (a) Western blot analysis of BHK cells transfected with either purified VPg-linked MNV RNA (vRNA) or plasmids expressing the cDNA encompassing the MNV subgenomic RNA [pT7 : MNV-SG (SG)], genomic RNA [pT7 : MNV-G (G)], genomic RNA containing a frame-shift mutation in the region coding for NS7 [pT7 : MNV-G^FS^ (G^FS^)] or the repaired derivative [pT7 : MNV-G^FS/R^ (G^FS/R^)]. Samples where infectious virus was recovered are indicated by +. (b) RT-PCR analysis demonstrating the presence of nuclease-resistant MNV RNA in nuclease-treated supernatants. PCRs were carried out with and without the prior addition of reverse transcriptase (RT). Positive and negative controls for PCR amplification (PCR− and PCR+) contained nuclease-free water or a plasmid encoding the MNV genomic RNA, respectively. Size of molecular mass markers is indicated.

Given our observation that FPV-T7 had no deleterious effect on MNV replication, yet was able to drive efficient expression of MNV proteins, the ability of FPV-T7 to allow the recovery of genetically defined norovirus in tissue culture was examined (Fig. 3). FPV-T7-infected cells were transfected with a cDNA construct containing the full-length MNV genomic cDNA (pT7 : MNV-G; Fig. 1b[Fig f1]), a similar construct that contained a frame-shift mutation in the viral RNA-dependent RNA polymerase NS7 (pT7 : MNV-G^FS^; Fig. 1b[Fig f1]) or a derivative of this construct in which the frame-shift mutation had subsequently been repaired (pT7 : MNV-G^FS/R^). To examine the requirement for the viral capsid proteins VP1 and VP2, cells were co-transfected with either empty vector or a cDNA construct containing the MNV subgenomic RNA (pT7 : MNV-SG). Western blot analysis demonstrated that high levels of NS7 were detected in cells transfected with either VPg-linked viral RNA or pT7 : MNV-G (Fig. 3a[Fig f3]). As predicted, a truncated NS7, referred to as NS7′, was detected in cells transfected with pT7 : MNV-G^FS^ (Fig. 3a[Fig f3]), whereas full-length NS7 was detected in cells that had been transfected with a construct in which the frame-shift mutation had been repaired (pT7 : MNV-G^FS/R^; Fig. 3a[Fig f3]). The viral capsid proteins VP1 and VP2 were only detected in cells transfected with the subgenomic cDNA expression construct pT7 : MNV-SG [Fig. 3(a) for VP1; data not shown for VP2].

As a first indication of the presence of encapsidated MNV RNA, the generation of nuclease-resistant MNV RNA was examined by RT-PCR of lysates from transfected cells that had been treated extensively with nucleases. Nuclease-resistant genomes were present in samples from cells transfected with VPg-linked viral RNA or a combination of MNV genomic and subgenomic cDNA constructs. To confirm that the production of encapsidated, nuclease-resistant viral RNA was the result of authentic virus replication by the viral RNA-dependent RNA polymerase NS7, the effect of a frame-shift mutation in NS7 on the production of nuclease-resistant viral RNA was examined (Fig. 3b[Fig f3]). Nuclease-resistant RNA was not detected in the lysates from cells transfected with a cDNA construct containing a frame shift in NS7 (pT7 : MNV-G^FS^), but was detected readily when cells were transfected with an identical construct in which the frame shift had been repaired (pT7 : MNV-G^FS/R^; Fig. 3b[Fig f3]).

The presence of infectious virus was subsequently confirmed by the ability to passage infectivity repeatedly to RAW 264.7 murine macrophages and confirmed by Western blot analysis of infected cells (data not shown). Infectious virus was reproducibly produced only from cells that had been transfected with purified viral RNA or transfected with cDNA constructs for both the full-length genomic and subgenomic RNAs (Fig. 3a[Fig f3]). As expected, a frame-shift mutation in NS7 prevented the recovery of infectious virus, which was subsequently recovered when the mutation was repaired (Fig. 3a[Fig f3]).

### Recovery of genetically tagged MNV

**Fig. 4. f4:**
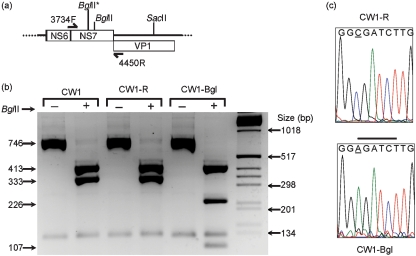
Recovery of genetically marked MNV. (a) Schematic representation of the mutated region of the MNV genome. The additional *Bgl*II restriction site introduced in the recombinant virus CW1-Bgl is indicated by an asterisk. (b) RT-PCR amplification and subsequent digestion of the amplified region, indicating the presence of an additional *Bgl*II site in the mutated virus CW1-Bgl that is absent in the parental recombinant CW1 virus derived from cDNA (CW1-R) or tissue culture-adapted MNV (CW1). Size of molecular mass markers is indicated (in bp). (c) Sequencing chromatogram of the RT-PCR products encompassing the mutated region of CW1 and CW1-Bgl. The nucleotide change introduced in CW1-Bgl is underlined; the introduced *Bgl*II site is indicated by a black line.

To confirm that the reverse-genetics system allowed the recovery of genetically defined noroviruses, an additional *Bgl*II restriction site was introduced into the MNV genome within the region encoding NS7. A single-nucleotide change (C to A) at position 3959 resulted in the introduction of a *Bgl*II site (Fig. 4a[Fig f4]). Recombinant viruses were recovered from cells co-transfected with a cDNA expression construct for the viral subgenomic RNA (pT7 : MNV-SG) along with either the wild-type full-length genomic cDNA construct (pT7 : MNV-G) or an identical construct containing the additional *Bgl*II restriction site (pT7 : MNV-G/*Bgl*II). The recovered viruses, referred to as CW1-R and CW1-Bgl, were subsequently amplified in tissue culture, and RT-PCR analysis of the region encoding NS7 was performed by using primers 3734F and 4450R. *Bgl*II digestion of the 746 bp amplified products demonstrated the presence of a single *Bgl*II site in the amplicons from the parental and recombinant MNV, CW1 and CW1-R (Fig. 4b[Fig f4]). However, an additional *Bgl*II site was present in the amplicon generated from the *Bgl*II-tagged virus, as evident by the generation of a 107 bp fragment (CW1-Bgl; Fig. 4b[Fig f4]). Direct sequencing of the amplicons confirmed the presence of the additional *Bgl*II site (Fig. 4c[Fig f4]). The entire sequence of the recovered viruses was determined by RT-PCR combined with 5′ and 3′ RACE, and no additional nucleotide changes were observed in either CW1-R or CW1-Bgl.

**Fig. 5. f5:**
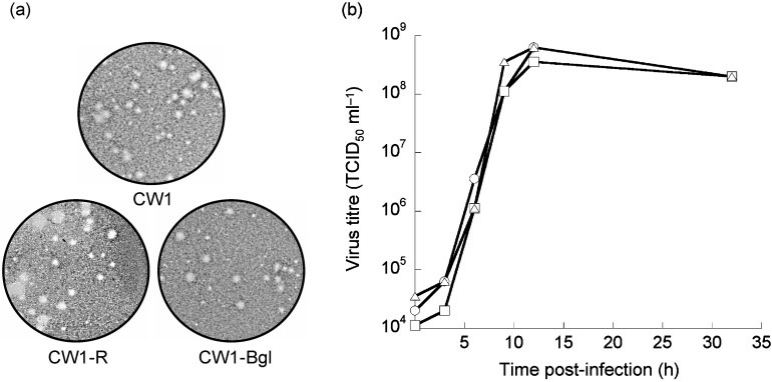
Growth characteristics of noroviruses recovered from cDNA. Plaque phenotype (a) and one-step growth-curve analysis (b) of wild-type MNV (CW1, ○), MNV recovered entirely from cDNA (CW1-R, □) and the recombinant MNV containing the additional *Bgl*II site (CW1-Bgl, ▵).

The phenotype of the viruses recovered entirely from cDNA (CW1-R and CW1-Bgl) was subsequently compared with that of the parental MNV CW1 by examining both plaque size and growth characteristics (Fig. 5). The growth characteristics of CW1-R and CW1-Bgl were identical to those of the parental virus CW1 (Fig. 5).

### The 3′-terminal nucleotide of the MNV genome is critical for efficient recovery

Our initial results indicated that recovery of MNV from cDNA required the expression of both the viral genomic and subgenomic RNAs (Fig. 3). However, during the course of the study, it became apparent that the 3′-terminal nucleotide immediately upstream of the poly-A tail in the cDNA expression construct pT7 : MNV-G did not match the MNV 1 consensus sequence derived from the mouse brain homogenate (GenBank accession no. AY228235). The terminal nucleotide directly upstream of the poly-A tail in all MNV strains sequenced to date is thymidine (T); however, in the cDNA construct pT7 : MNV-G, the terminal nucleotide was cytosine (C) (Fig. 1b[Fig f1]). Sequence analysis of the subgenomic RNA expression construct indicated that it matched the MNV consensus in that the terminal nucleotide was a thymidine (Fig. 1b[Fig f1]). The sequence of both CW1-R and CW1-Bgl indicated that, in each case, the terminal nucleotide matched the MNV consensus sequence, namely thymidine (data not shown). To determine whether the apparent requirement for the subgenomic RNA was due to the 3′-terminal mutation in the genomic RNA construct, the mutation was repaired to generate the construct pT7 : MNV-G 3′Rp and an additional construct was generated in which a hepatitis delta virus ribozyme was inserted after the poly-A tail (pT7 : MNV-G 3′Rz; Fig. 1b[Fig f1]). Results indicated that high levels of NS7 were produced from these constructs, but that the viral capsid proteins VP1 and VP2 were undetectable by Western blot unless cells were co-transfected with a subgenomic RNA expression construct (data not shown). Interestingly, despite the undetectable levels of the capsid proteins, infectious virus was generated when cells were transfected with the constructs containing the correct 3′-terminal nucleotide, pT7 : MNV-G 3′Rp or pT7 : MNV-G 3′Rz, alone (Table 1). Our studies also indicated that the inclusion of a hepatitis delta virus ribozyme at the 3′ end of the viral genome increased the efficiency with which virus was recovered (Table 1). Infectious virus was only detected 72 h post-transfection when cells were co-transfected with the subgenomic RNA expression construct along with the construct containing an incorrect terminal nucleotide (pT7 : MNV-G; Table 1). However, infectious virus was detected 24 h post-transfection if cells were transfected with constructs containing the correct 3′-terminal nucleotide, pT7 : MNV-G 3′Rp or pT7 : MNV-G 3′Rz (Table 1). The yield of virus was greater when a hepatitis delta virus ribozyme was included at the 3′ end of the viral genome (Table 1), suggesting that a free 3′ end may be required for efficient virus recovery.

### Recovery of noroviruses from different cell types

Our work described above relied exclusively on the use of the BHK (baby hamster kidney) cell line, due to its high efficiency of transfection and our observation that transfection of purified viral RNA resulted in the production of >10^6^ TCID_50_ from a 35 mm dish (Fig. 2a[Fig f2]). Hence, this would indicate that all of the necessary intracellular factors required for MNV replication are present in hamster cells, despite our observation that they cannot be infected (data not shown). Several attempts to recover MNV from cDNA using the RAW 264.7 cell line, which is permissive to viral infection, failed to lead to the production of infectious virus. This was probably due to the low efficiency of transfection (data not shown). To begin to optimize and increase the efficiency of virus recovery, the yield of virus from a number of different cell lines was examined following transfection with the infectious clone containing a correct 3′-terminal nucleotide and a 3′ ribozyme (Table 2). The BHK cell line and a derivative modified to express T7 RNA polymerase (BSR-T7) were found to produce the highest yields of virus (Table 2), on average approximately 3.36×10^4^ TCID_50_ per 35 mm dish. The BSR-T7 cell line was examined as a potential method of recovering virus in the absence of FPV-T7 infections; however, in all cases, infectious virus was only obtained when cells had previously been infected with FPV-T7. The Huh 7.5 cell line, previously reported to be deficient for the antiviral sensor Rig-I (Sumpter *et al.*, 2005), was also examined and was also found to yield relatively low levels of virus from cDNA (approx. 2.4×10^4^ TCID_50_ per 35 mm dish; Table 2). The typical yield from 293T cells was approximately 6×10^3^ TCID_50_ per 35 mm dish (Table 2). Transfection of the majority of cells examined with VPg-linked viral RNA resulted in the production of >10^7^ TCID_50_ per 35 mm dish (Table 2), indicating that all cells examined were capable of efficient MNV replication. Vero cells, defective for interferon production (Emeny & Morgan, 1979), yielded relatively low levels of virus when transfected with VPg-linked viral RNA (approx. 1.9×10^5^ TCID_50_ per 35 mm dish; Table 2) and no virus was recovered by using the reverse-genetics system (Table 2). This may reflect a low transfection efficiency or the effect of the increased cytopathic effect that FWPV infection appeared to cause in this cell line (data not shown).

## DISCUSSION

In this report, we describe the generation of the first norovirus reverse-genetics system, using an FWPV recombinant to deliver T7 RNA polymerase. Previously described reverse-genetics systems for FCV (Sosnovtsev *et al.*, 2002; Thumfart & Meyers, 2002) have relied on the use of a VACV recombinant to drive T7 RNA polymerase expression; however, our studies indicated that VACV infection had a negative effect on the replication of MNV. The first T7 RNA polymerase recombinant VACV, vTF7-3 (Fuerst *et al.*, 1986), was based on strain WR and was thus fully replication-competent, i.e. capable of lytic replication in a range of mammalian and animal cell types. Like the parental virus, vTF7-3 shuts down most host-gene expression, but proved extremely useful for high-level expression of foreign genes. However, presumably because of its cytopathic nature, it proved inhibitory to the replication of some viruses and has been less useful for the recovery of RNA viruses from full-length cDNA clones. T7 RNA polymerase recombinants of the host range-restricted VACV MVA were therefore developed (Sutter *et al.*, 1995; Wyatt *et al.*, 1995). Restricted for productive replication to avian cells and just a few mammalian cell lines, these recombinants are less lytic in most cell lines (although they can still induce cytopathic effects, especially at high m.o.i.). As a consequence, these recombinants have been used successfully to recover several RNA viruses from full-length cDNA clones, including a vaccine strain of rinderpest virus (Baron & Barrett, 1997), measles virus (Schneider *et al.*, 1997), Sendai virus (Leyrer *et al.*, 1998), mumps virus (Clarke *et al.*, 2000), canine distemper virus (Gassen *et al.*, 2000), bovine respiratory syncytial virus (Yunus *et al.*, 2001) and FCV (Thumfart & Meyers, 2002). MVA is, however, highly cytopathic for some cells and cell lines, including many primary mammalian (but not primate) cells (Das *et al.*, 2000). Where such cells are required for propagation of, for instance, field strains of rinderpest virus, the MVA-induced cytopathic effect appears to compromise T7 RNA polymerase-mediated virus recovery (Das *et al.*, 2000). Under such circumstances, FPV-T7 has often proved capable of virus rescue (Boot *et al.*, 1999; Peeters *et al.*, 1999; Das *et al.*, 2000; Casais *et al.*, 2001). Even in cells in which both MVA and FWPV can replicate (i.e. avian cells only) or in cells in which both MVA and FWPV cause cytopathic effects, FPV-T7 can often still rescue when MVA cannot, probably because of the slower replication dynamics of FWPV compared with MVA.

Also worth noting is the fact that the T7 RNA polymerase in the MVA-T7 used in this study is under the control of a late (p11) poxvirus promoter (Wyatt *et al.*, 1995), whereas that in FPV-T7 is under the control of the early/late poxvirus promoter, p7.5 (Britton *et al.*, 1996). It is conceivable that the use of an MVA-T7 expressing T7 RNA polymerase from an early/late promoter (Sutter *et al.*, 1995) might have proved capable of rescuing MNV. However, the observation that equivalent levels of MNV proteins were expressed from pT7 : MNV-G in cells infected by either MVA-T7 or FPV-T7 argues somewhat against this. The inhibitory effect of MVA-induced cytopathic effect on expression from VPg-linked RNA might have been countered by the use of cytosine arabinoside, which blocks MVA genome replication. Such a strategy was used successfully to rescue measles virus (Kovacs *et al.*, 2003), but again, it would have required the use of an MVA with T7 polymerase under the control of an early or early/late promoter.

Our inability to recover MNV by transfection of *in vitro*-transcribed, capped or uncapped RNA is in contrast to previous reports on FCV (Sosnovtsev & Green, 1995; Thumfart & Meyers, 2002), PEC (Chang *et al.*, 2005) and RHDV (Liu *et al.*, 2006). It is probable that this simply reflects the poor stability of the RNA or the inability of the host-cell translation machinery to translate capped or uncapped MNV RNA efficiently. Indeed, our previous work on calicivirus translation would indicate that VPg is required for efficient translation of calicivirus RNA (Goodfellow *et al.*, 2005; Chaudhry *et al.*, 2006). We have also demonstrated that, despite the ability of MNV VPg to interact with eIF4E, VPg-linked MNV viral RNA is able to translate in the absence of eIF4E *in vitro* (Chaudhry *et al.*, 2006). This indicates that translation initiation on MNV RNA requires a subset of initiation factors different from that required by capped host-cell mRNAs. Our unpublished data would confirm that *in vitro* translation of capped or uncapped MNV RNA is inefficient compared with that of VPg-linked viral RNA (data not shown).

During our study, we also examined the ability of the BSR-T7 cell line, a BHK cell line derivative that expresses T7 RNA polymerase constitutively, to allow the recovery of MNV in the absence of FWPV infection (Table 2). This cell line was chosen due to the reported success in recovery of respiratory syncytial virus (Buchholz *et al.*, 1999) and bunyaviruses (Lowen *et al.*, 2004), as well as a potential method of helper-free virus recovery. However, we failed to detect MNV protein expression after transfection of the BSR-T7 cell line with MNV cDNA constructs (data not shown). Protein expression and infectious virus were only detected after prior infection with FPV-T7 (data not shown). This may be due to low levels of RNA synthesis in these cells in the absence of FPV-T7 infection or, as described above, to the fact that the uncapped MNV transcripts produced in this cell line are either unstable or poorly translated. Although previous reports would suggest that between 5 and 10 % of the transcripts produced by T7 RNA polymerase in VACV-infected cells are capped (Fuerst & Moss, 1989), similar studies have not been carried out for FPV-T7. It is likely that some of the transcripts produced by using the FPV-T7 system are capped by the fowlpox capping enzymes; however, future studies would be required to confirm this.

Given the previously reported role of interferon in controlling calicivirus replication (Karst *et al.*, 2003; Chang *et al.*, 2004; Wobus *et al.*, 2004), we examined the recovery of virus by using the reverse-genetics system from cell lines defective for various aspects of the interferon response (Vero and Huh 7.5 cells). However, the yield of virus from these cell lines was no greater than that obtained from cells in which the interferon system was intact (Table 2). In fact, the Vero cell line, defective for interferon synthesis (Emeny & Morgan, 1979), failed to recover any infectious virus and appeared to replicate MNV to only low levels. Further studies also demonstrated that expressing the V protein from simian virus 5, which is known to lead to the degradation of STAT-1 (Andrejeva *et al.*, 2002), had no stimulatory effect on MNV recovery in 293 cells (data not shown). Hence, our data would indicate that the interferon system plays no role in the restriction of virus recovery using the reverse-genetics system, although further studies are warranted.

Our results would also suggest that the sequence of the 3′-terminal nucleotide of the MNV genome is critical for efficient recovery, as constructs containing an incorrect 3′-terminal nucleotide required the co-expression of the MNV subgenomic RNA for recovery (Fig. 3a[Fig f3]; Table 1). Interestingly, viruses recovered by using the genomic RNA construct that contained an incorrect 3′-terminal nucleotide appeared to correct the 3′-terminal nucleotide to match that present in all previously sequenced MNV strains (thymidine; data not shown). It is possible that the repair of the 3′-end defect was the result of low-frequency recombination between the genomic and subgenomic RNAs; however, further studies would be required to confirm this. The inclusion of a hepatitis delta ribozyme at the 3′ end of the viral genome was found to increase virus yield and resulted in maximal virus production 24 h post-transfection (Table 2), possibly indicating that a free 3′ end is required for efficient virus replication.

Seroprevalence studies have highlighted that, as well as functioning as a model for the human noroviruses, MNV is a significant pathogen in its own right, with 22 % of mouse colonies in the USA and Canada found to be seropositive (Hsu *et al.*, 2005). Recent studies have also highlighted the fact that numerous strains appear to circulate, which can differ markedly in their ability to replicate and cause disease in the host (Hsu *et al.*, 2006; Mumphrey *et al.*, 2007). As more strains are identified, by combining sequence data, the mouse model and the reverse-genetics system described herein, it will now be possible to correlate sequence variation with differences in norovirus pathogenicity. Undoubtedly, such studies will facilitate the identification of viral and host-cell factors required for norovirus pathogenesis and will aid our ability to control members of this economically important family of viruses.

## Supplementary Material

[Supplementary table]

## Figures and Tables

**Table 1. t1:** Efficiency of recovery of MNV All cells were infected with FPV-T7 prior to transfection. See Methods for details of the constructs used. Virus titre was determined at 24 and 72 h post-transfection and expressed as TCID_50_ per 35 mm dish. −, No detectable virus was generated.

**Construct**	**Virus yield at indicated time post-transfection**	
	**24 h**	**72 h**
pT7 : MNV-G	−	−
pT7 : MNV-G+pT7 : MNV-SG	2.00×10^2^	2.00×10^3^
pT7 : MNV-G 3′Rp	2.00×10^3^	6.32×10^3^
pT7 : MNV-G 3′Rp+pT7 : MNV-SG	6.32×10^3^	3.56×10^3^
pT7 : MNV-G 3′Rz	3.56×10^4^	1.12×10^4^
pT7 : MNV-G 3′Rz+pT7 : MNV-SG	1.12×10^4^	3.56×10^3^

**Table 2. t2:** Yield of MNV from various cell lines All cells were infected with FPV-T7 prior to transfection to control for any effects of FPV infection on MNV replication. Cells were then transfected with purified VPg-linked viral RNA (vRNA) or the MNV infectious clone (pT7 : MNV-G 3′Rz). Virus yield was determined 24 h post-transfection and expressed as TCID_50_ per 35 mm dish. Assays were performed a minimum of three times and representative data from one experiment are shown. −, No detectable virus was generated.

**Cell type**	**vRNA**	**pT7 : MNV-G 3′Rz**
BHK	1.00×10^7^	3.36×10^4^
BSR-T7	6.00×10^7^	3.36×10^4^
293T	3.36×10^7^	6.00×10^3^
Huh 7.5	6.00×10^7^	2.40×10^4^
Vero	1.90×10^5^	−
