# Supplementary material for: Recovery of genetically defined murine norovirus in tissue culture by using a fowlpox virus expressing T7 RNA polymerase
Source: J Gen Virol. 2007 Aug;88(Pt 8):2091–100. doi: 10.1099/vir.0.82940-0 (PMC2884977; doi:10.1099/vir.0.82940-0)
Supplement: [Supplementary table] [file supp_88_8_2091__index.html]

 Recovery of genetically defined murine norovirus in tissue culture by using a fowlpox virus expressing T7 RNA polymerase -- Chaudhry et al. 88 (8): 2091 Data Supplement - Supplementary table -- Journal of General Virology

### Recovery of genetically defined murine norovirus in tissue culture by using a fowlpox virus expressing T7 RNA polymerase, by Y. Chaudhry, M. A. Skinner and I. G. Goodfellow

*Journal of General Virology* vol. **88**, part 8, pp. 2091 – 2100

**Supplementary Table S1.** Oligonucleotides used during this study [PDF] (64 KB)

  
  
