# Supplementary material for: Recovery of genetically defined murine norovirus in tissue culture by using a fowlpox virus expressing T7 RNA polymerase
Source: J Gen Virol. 2007 Aug;88(Pt 8):2091–100. doi: 10.1099/vir.0.82940-0 (PMC2884977; doi:10.1099/vir.0.82940-0)
Supplement: [Supplementary table] [file supp_88_8_2091__1.pdf]

Supplementary Table S1. Oligonucleotides used during this study

Name	Sequence (5′→3′)
IGIC21	ATAGTTTAGCGGCCGCTAATACGACTCACTATAGTGAATGAGGATGAGTGATGGCGCAGCGCC
IGIC22	AACCCATTTAAATTTTTTTTTTTTTTTTTTTTTTTTTTAAAATGCATCTAACTACCACAAAGAAAAGAAAGC
IGIC37	TCGCGAGCTAGCTTTTTTTTTTTTTTTTTTTTTTTTTTAAAATGCATCTAACTACCACAAAGAAAAGAAAGC
IGIC44*	GGACTGGACGGGGAGCGCTTTTATTGGAGATCTTGGTGACCAGGCCACCC
3734F	GCGAGATCAGCTTAAGCCCTATTCAGAACCACGCG
4450R	CCAAACTATTCAGCTGTGTGGTGCAAGGGC
7155F	GTGGACACATCCCCTCTACCGATCTCGGGTGGACGCTTGCC
7400R	TTTTTTTTTTTTTTTTTTTTTTTTTTTTTTAAAATGCATCTAACTACCACAAAG
PUCF	CCCAGTCACGACGTTGTAAAACG
PUCR	ACACAGGAAACAGCTATGACCA

*The introduced *Bgl*II site is underlined.

Chaudhry, Y., Skinner, M. A. & Goodfellow, I. G. (2007). Recovery of genetically defined murine norovirus in tissue culture by using a fowlpox virus expressing T7 RNA polymerase. *J Gen Virol* **88**, 2091–2100.
